# Mouse strain specific gene expression differences for illumina microarray expression profiling in embryos

**DOI:** 10.1186/1756-0500-5-232

**Published:** 2012-05-14

**Authors:** Petra Kraus, Xing Xing, Siew Lan Lim, Max E Fun, V Sivakamasundari, Sook Peng Yap, Haixia Lee, R Krishna Murthy Karuturi, Thomas Lufkin

**Affiliations:** 1 Stem Cell and Developmental Biology, Genome Institute of Singapore, Singapore, 138672, Singapore; 2 Computational and Systems Biology, Genome Institute of Singapore, Singapore, 138672, Singapore

**Keywords:** Microarray, Mouse strain, Polymorphism, Expression profiling, Transgenic mice

## Abstract

**Background:**

In the field of mouse genetics the advent of technologies like microarray based expression profiling dramatically increased data availability and sensitivity, yet these advanced methods are often vulnerable to the unavoidable heterogeneity of *in vivo* material and might therefore reflect differentially expressed genes between mouse strains of no relevance to a targeted experiment. The aim of this study was not to elaborate on the usefulness of microarray analysis in general, but to expand our knowledge regarding this potential “background noise” for the widely used Illumina microarray platform surpassing existing data which focused primarily on the adult sensory and nervous system, by analyzing patterns of gene expression at different embryonic stages using wild type strains and modern transgenic models of often non-isogenic backgrounds.

**Results:**

Wild type embryos of 11 mouse strains commonly used in transgenic and molecular genetic studies at three developmental time points were subjected to Illumina microarray expression profiling in a strain-by-strain comparison. Our data robustly reflects known gene expression patterns during mid-gestation development. Decreasing diversity of the input tissue and/or increasing strain diversity raised the sensitivity of the array towards the genetic background. Consistent strain sensitivity of some probes was attributed to genetic polymorphisms or probe design related artifacts.

**Conclusion:**

Our study provides an extensive reference list of gene expression profiling background noise of value to anyone in the field of developmental biology and transgenic research performing microarray expression profiling with the widely used Illumina microarray platform. Probes identified as strain specific background noise further allow for microarray expression profiling on its own to be a valuable tool for establishing genealogies of mouse inbred strains.

## Background

Mouse models are a fundamental tool in gaining a better understanding of mammalian development in general and human pathology in particular [1]. By studying gene expression patterns in the developing mouse embryo, important genetic pathways and signaling cascades have been revealed, for example in limb patterning (for review see [2]), the central nervous system (for review see [3])and the digestive system (for review see [4]). However, gene expression profiling techniques have leaped forward in recent years from the classical RNA in situ hybridization analysis to the more detailed and advanced methodologies of microarray analysis [5-8] providing a powerful tool for in depth analysis of genome wide differential gene expression. Yet, by increasing the assay sensitivity and being able to detect more subtle changes in expression profiles, questions arise in how far differences in the mouse genetic background affect the outcome of this advanced type of gene expression profiling. It is of concern, that strain specific differences in gene expression levels might obscure the readout of microarray results for certain genes or tissue types simply by reflecting “background noise” resulting either from true genetic strain dependent differences in expression levels yet, not relevant for a more targeted study or from a “chip artifact” originating from unfavorable hybridization conditions of some probes due to polymorphisms in their DNA sequence [7,9,10].

Microarray data is often obtained from cell culture approaches with a fairly homogenous genetic background [11,12], these however lack the native context provided by *in vivo* assays analyzing fresh adult tissue or embryos. Historically, the mouse has often been the model organism of choice for *in vivo* studies and it is well known that different mouse inbred strains differ in their behavioral traits, physiology and anatomy [1,9,13-16]. Extensive data has been generated thus far addressing differential gene expression, especially for the Affymetrix array platform, mostly focusing on adult tissue often of the sensory and central nervous system (CNS) type, frequently restricted to only one tissue type or a couple of inbred strains selected for their suitability in behavior studies or within one strain at different time points [7-9,17-21]. More recent *in vivo* approaches however combine transgenic models with tissue dissection and microarray based gene expression profiling [5,22,23]. Modern genetic engineering often requires crosses between several mouse strains, for example by breeding mice harboring a targeted allele to Flpe- or Cre-deleter strains, yet the production of isogenic strains for each genetically modified allele generated would exceed most funding time frames [24-28].

When studying prenatal development availability of sufficient material can be another limiting factor for expression profiling, hence the breeding advantage of hybrid or outbred strains is often considered [29-32] ( http://www.harlan.com). Despite a vast amount of existing data (for a review see [10]), it remains crucial for studies making use of genetically engineered animals to expand our current knowledge of gene expression profiling background noise to additional inbred and even outbred strains and also to a spectrum of embryonic time points, ideally for all microarray platforms as the outcome of expression profiling is clearly dependent on the platform used [7,33,34].

With the ultimate aim to complement existing data, using the Illumina microarray platform, we performed a comparative analysis across several commonly used mouse strains in transgenic research (C57BL/6J, 129 S2/SvHsd, FVB/NHan^TM^Hsd and Hsd:ICR(CD-1)®) at three different stages of mid-gestation development and an additional comprehensive comparison across 11 strains [129 S2/SvHsd, FVB/NHan^TM^Hsd, C3H/HeNHs, CBA/JHsd; BALB/cOlaHsd, C.B-17/IcrHan^TM^Hsd-*Prkdc*^*scid*^, C57BL/6 J, B6;SJL-Tg(Col2a1-cre)1Bhr/J, 129 S4/SvJaeSor-*Gt(Rosa)26Sor*^*tm1(FLP1)Dym*^/J, C57BL/6J(Zp3-cre)93Knw/J, Hsd:ICR(CD-1)®] at E12.5 focusing on eviscerated embryos to provide a reference list of gene expression differences, while at the same time observing the impact of a reduction in tissue diversity and increasing genetic strain diversity on differential gene expression levels.

## Results and discussion

### A reference resource for gene expression profiling associated background noise with the illumina mouse WG6 v2.0 microarray in transgenic research and developmental biology

Long lists of up and down regulated genes are the general outcome of microarray based differential gene expression analysis, often with genes of unknown function or misleading gene ontology (GO) terms. Factors like polymorphisms in the genetic background are known to impact on the interpretation of differential expression profiling data sets [35]. This problem is faced when comparing different species like primate and non-primate [36,37] or different mouse inbred strains [8] and expected when applying differential expression profiling to material from genetically engineered and often still non-isogenic animals or embryonic tissue. In order to generate a reference of gene expression profiling-associated background noise in wild type embryos of strains typically used for gene modification, we chose three *Mus musculus musculus* inbred strains most commonly used in developmental genetics, gene targeting or transgenic mouse production procedures (C57BL/6J, 129 S2/SvHsd, FVB/NHan^TM^Hsd) along with an outbred mouse strain Hsd:ICR(CD-1)® to address the differential gene expression profile of entire embryos at three mid-gestation developmental stages (E11.5, E12.5 and E13.5), asking the question: Is there a significant strain specific difference for any probe at any given time point? The approach of embryo-pooling according to the experimental design of Korostynski et al. [20] was chosen to generate four biological replicates for each strain and stage analyzed while minimizing the contribution of individual differences or slightest technical variations to the differential expression profile (for details refer to Experimental Design in the Material and Methods section).

According to Illumina’s probe list the Mouse WG-6_V2_0_R3_11278593 array contains a total of 45282 probe sequences and is based on a C57BL/6J genome. Many of the probes on the array are unique, while some loci are represented by multiple probes. Of all these 45282 probes subjected to differential gene expression analysis transcripts of a total of 580 probes (1.28%) at E11.5, 503 probes (1.11%) at E12.5 and 836 probes (1.85%) at E13.5 were found to be significantly differentially regulated when subjecting entire wild type embryos to this strain specific gene expression profiling (for a full list see Additional file 1: Tables S1, Additional file 2: Table S2 and Additional file 3: Table S3). Some of the probes were found ranking in the top 20 for all three stages examined: *Fcer1g*, *Lrrc57*, *Sspn*, *Tmem87a*, *Cap1*, *Lip1*, *Gramd4*, *Ctse*, *Tm7sf3*, *Pou6f1*, *LOC382555* (*Hmgb1-rs18*) and *C920006O11Rik* (unclassified RIKEN cDNA) (for details see Additional file 1: Tables S1, Additional file 2: Table S2 and Additional file 3: Table S3, for details regarding the probe ranking refer to Experimental Design in the Material and Methods section.)

The fact that many genes are represented by only a single oligo probe on the array increases the risk that differential expression among the four strains, particularly if observed for a given strain at all three stages (for example in the case of *Fcer1g* and *Lrrc57*) results from strain dependent polymorphisms affecting the probe-mRNA hybridization efficiency and causing misleading expression profiles. To examine this, we sequenced over the genomic region of all four strains analyzed in this study including and flanking the region of a few randomly selected probes showing consistent strain differences, namely: *Sspn*, *Lrrc57*, *Fcer1g*, *Zfp235*, *Gramd4*, *Cfl1*, *Pou6f1*, *Snx5*^*b*^, *Lib1* and *Lip1*^*b*^. The genomic sequences of *Fcer1g*, *Lrrc57* and *Gramd4* indeed show polymorphisms compared to the oligo sequence represented on the Illumina MouseWG-6_V2_0_R3_11278593 array for the strains 129 S2/SvHsd, FVB/NHan^TM^Hsd and Hsd:ICR(CD-1)® but not for C57BL/6J supporting the observation of a higher average signal for embryos of the C57BL/6J strain, owing to less favorable hybridization conditions for the other strains. For *Pou6f1*, *Snx5*^*b*^ and *Zfp235* on the other side, polymorphisms were only detected for the 129 S2/SvHsd strain, explaining the generally lower average signal observed for embryos from this strain. Similarly, lower average signals in embryos from the FVB/NHan^TM^Hsd strain for *Lip1* could be attributed to polymorphisms in both probes on the array (for details please refer to Table 1). The polymorphisms and resulting hybridization disadvantages rather than true differential expression are likely responsible for the noted strain specific differences in expression levels at a given developmental stage. In the case of *Sspn*, one of our top ranking probes, (for details regarding the probe ranking refer to Experimental Design in the Material and Methods section), polymorphisms and deletions found for the Hsd:ICR(CD-1)® and the FVB/NHan^TM^Hsd strains explain the lower expression observed for these two strains compared to embryos of C57BL/6J and 129 S2/SvHsd background (see Table 1). Hence all randomly selected and sequenced probes in our study displaying strain specific differential gene expression could be labeled as “background noise” based on the identification of strain specific polymorphisms, yet for *Cfl1*or *cofilin 1* (MGI:101757), a single transcript gene, sequencing did not reveal any polymorphisms for any of the four strains in the region overlapping with the Illumina probe. In an attempt to understand whether strain specific gene expression indeed could account for the differential expression profile noted for *Clf1*, we performed for this one gene quantitative PCR validation (qPCR) for two primer sets probing for the *Cfl1* transcript on the same total mRNA pool previously subjected to the array. Since the qPCR results did not support our array data (data not shown), we mapped the exact genomic localization of the *Cfl1* oligo spotted on the array and found it to be located outside the actual *Cfl1* gene. According to NCBI mapview the only two genes in the vicinity, *Sorting nexin 32* (*Snx32*, MGI:2444704) and *Clf1* itself do not overlap with the probe spotted on the array. Since our input source was total mRNA likely including unedited nuclear RNA and based on the transcriptional direction of *Snx32* and *Clf1* relative to the array probe only an unedited *Clf1* transcript could have hybridized with the so called “*Cfl1* probe” on the array. It is of note that according to Illumina both the MouseWG-6 v2.0 array platform used in this study ( http://www.illumina.com/products/mousewg_6_expression_beadchip_kits_v2.ilmn) as well as the previous version MouseRef8 v2.0 ( http://www.illumina.com/products/mouseref-8_v2_expression_beadchip_kit.ilmn) are derived from the National Center for Biotechnology Information Reference Sequence (NCBI RefSeq) database (Build 36, Release 22). The MouseWG-6 v2.0 array is said to contain all probes of the MouseRef8 v2.0 array with an additional 11,603 probes from the Mouse Exonic Evidence Based Oligonucliotide (MEEBO) as well as exemplar protein-coding sequences described in the RIKEN FANTOM2 database. The build used for both array platforms likely differs in gene annotation from the currently available NCBI RefSeq database Build 37. The lack of qPCR validation for *Clf1* could hence be due to a probe design artifact which itself might be attributed to updates in gene annotation. Given that despite this artifact we still detected the “*Clf1*” probe as a strain specific differentially expressed transcript might result from the fact that the probe detects various levels of unedited *Clf11* nuclear transcript.

**Table 1:** List of the top 50 targets ranked by total fold change following a strain by strain comparison after microarray based differential expression analysis for the following three data sets

**Position**	**E12.5 entire embryo/4 strains**	**E12.5 eviscerated embryos/4 strains**	**E12.5 eviscerated embryos/11 strains**
1	LOC382555	Tm7sf3	Tm7sf3
2	Gramd4	Fcer1g	Fcer1g
3	Sspn	LOC100041516	C920006O11Rik
4	LOC666403	Pou6f1	Lrrc57
5	EG384179	C920006O11Rik	scl0001602.1_506
6	Tmem87a	Smad2	LOC100041516
7	Cap1	Lrrc57	Pou6f1
8	C920006O11Rik	Mod1	Sspn
9	Lrrc57	Sspn	LOC382555
10	Fcer1g	Ctse	Mod1
11	Ctse	Map3k4	Smad2
12	Lip1	Gmfb	Thumpd1
13	Eif2s3y	AA388235	Myl2
14	6230403H02Rik	LOC382555	OTTMUSG00000010673
15	2810417H13Rik	Tmem87a	AA388235
16	Prkag2	Crygd	Tmem87a
17	Pdrg1	6230403H02Rik	Map3k4
18	B230312I18Rik	9430065F12Rik	LOC641366
19	Tm7sf3	LOC641366	Gmfb
20	Atrnl1	LOC100043918	Klk1b22
21	Pou6f1	Klk1b22	2610203C20Rik
22	Cbwd1	D930007N19Rik	9430065F12Rik
23	Plk1	B230213E18Rik	Ctse
24	Drbp1	Cfl1	Bat5
25	LOC100041516	LOC229810	EG631624
26	Paip1	1700001E04Rik	Bag2
27	Ube2i	Cryaa	3110099E03Rik
28	Itga9	EG631624	LOC229810
29	2310002F18Rik	Crygb	3110007F17Rik
30	Snx5	2600001B17Rik	6230403H02Rik
31	Rusc2	D330027H18Rik	6720422M22Rik
32	2810410P22Rik	BC032265	2600001B17Rik
33	Csrp1	3110003A22Rik	Rbm45
34	Zfp330	Cap1	3110003A22Rik
35	C920004C08Rik	Cryga	Usp25
36	1110005F07Rik	A630064P09Rik	Apol7c
37	LOC229810	Usp25	LOC100044289
38	EG666668	LOC100044289	Cryaa
39	Zcchc3	LOC100040657	B230213E18Rik
40	3110040M04Rik	LOC622901	LOC384154
41	Ttc27	2610203C20Rik	D330027H18Rik
42	Cops8	3110099E03Rik	Zfp35
43	Aph1b	Gramd4	EG384179
44	Prcp	LOC100048169	Cap1
45	Mff	LOC100044150	4930488E11Rik
46	Slc25a17	Serpina3h	Ccdc109b
47	Tor1aip2	6720422M22Rik	Rnf20
48	Adamts9	C130082I06Rik	Crygd
49	6720422M22Rik	EG384179	LOC666661
50	Lrp6	Rbm45	1700001E04Rik

**Rank A** (entire embryos of four strains (C57BL/6J, 129 S2/SvHsd, FVB/NHan ^TM^Hsd and Hsd:ICR(CD-1)® at E12.5), **Rank B** (eviscerated embryos of four strains (C57BL/6J, 129 S2/SvHsd, FVB/NHan ^TM^Hsd and Hsd:ICR(CD-1)® at E12.5) and **Rank C** (eviscerated embryos of 11 strains (129 S2/SvHsd; FVB/NHan ^TM^Hsd; C3H/HeNHs; CBA/JHsd; BALB/cOlaHsd; C.B-17/IcrHan^TM^Hsd-*Prkdc*^*scid*^; C57BL/6J; B6; SJL-Tg(Col2a1-cre)1Bhr/J; 129 S4/SvJaeSor-*Gt(Rosa)26Sor*^*tm1(FLP1)Dym*^/J; C57BL/6-Tg(Zp3-cre)93Knw/J; Hsd:ICR(CD-1)®) at E12.5.

**Figure 1:**
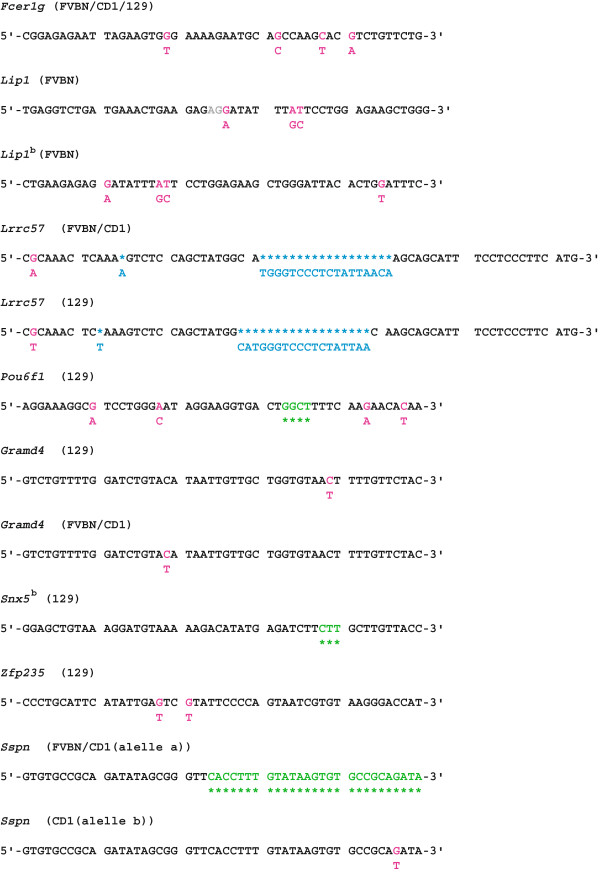
**List of selected targets showing a strain-dependent signal in two and more of the data sets analyzed.** Analyzed targets are italicized, affected strains follow in parenthesis and are abbreviated as following: 129 (129 S2/SvHsd), FVBN (FVB/NHan ^TM^Hsd) and CD1 (Hsd:ICR(CD-1)®)). Strain dependent signals could generally be attributed to polymorphisms with regards to the C57BL6 based Illumina probe as indicated: single nucleotide exchange (pink), insertion (blue) and deletion (green).

While less than 2% of the array appeared strain sensitive when subjecting entire embryos to the analysis, it is advisable to be cautious and to compare the array probe sequence of a potential target gene of interest with the genetic background of the source used for expression profiling as well as the actual genomic location of the probe to avoid any misinterpretation of the array data. A hypoxia study in CD-1 mice from Charles River by Zhou et al. [38] makes a point of *Sspn* being the only gene to be downregulated for all conditions and in all brain regions of their assay using the Sentrix mouse-6 expression bead chip from Illumina, which seemingly corresponds to the array used in our study http://expression.genomecenter.ucdavis.edu/illumina_expre.html. Notably, we have observed a lower average signal for *Sspn* for the Hsd:ICR(CD-1)® embryos in our study which could be attributed to point mutations and deletions between the Hsd:ICR(CD-1)® and C57BL6 genomic background (Figure 1). While the authors validate their findings with downstream cell culture experiments [38], it does demonstrate that there is need for caution, especially when working with strains that differ from the C57BL6 genetic background the Illumina arrays are based upon. While outbred lines like the frequently used Hsd:ICR(CD-1)® are often chosen for their breeding advantage, it needs to be considered that to maintain a random outbred line, breeding schemes are developed to avoid inbreeding in order to maintain a maximum level of heterozygosity. (For reference see [32]). The different genetic background allows for multiple polymorphisms within the strain, resulting in less predictable hybridization conditions between the C57BL6 derived probe and RNA generated from these outbred strains, hence it is of utmost importance to critically review microarray results when working with an outbred line.

Based on the use of different platforms, time points and tissues naturally the transcripts identified as differentially expressed are likely to differ for most datasets. Yet, members of the *Serpina* gene family appear for different types of analyses and tissues: *Serpina3n*, formerly known as *Spi2-2spi2/eb4* or M64086, was identified as a target using the Affymetrix platform in chondrogenesis [39] and in neural studies [9,21], while *Serpina3h* and *Serpina1e*, appeared in ranking 26 and 11 respectively for our differential expression analysis at E13.5 (see below) using the Illumina platform. All three genes belong to the clade A of serine (or cysteine) peptidase inhibitors according to the MGI database http://www.informatics.jax.org/mgihome/.

### Gene expression profiling with the illumina mouse WG6 v2.0 microarray chip on wild type mid-gestation embryos reflects known developmental patterns and has the potential to identify novel candidates

In an attempt to validate and further explore our microarray data we expanded our analysis from the gene expression profiling at each individual time point as described above to the three mid-gestation time points E11.5, E12.5 and E13.5, asking the following questions: Do genes group in a logical fashion according to their known expression patterns during development? And if so, can we discover new targets of potential interest simply based on similarities in their time course heat map to already well established and characterized genes?

**Figure 2:**
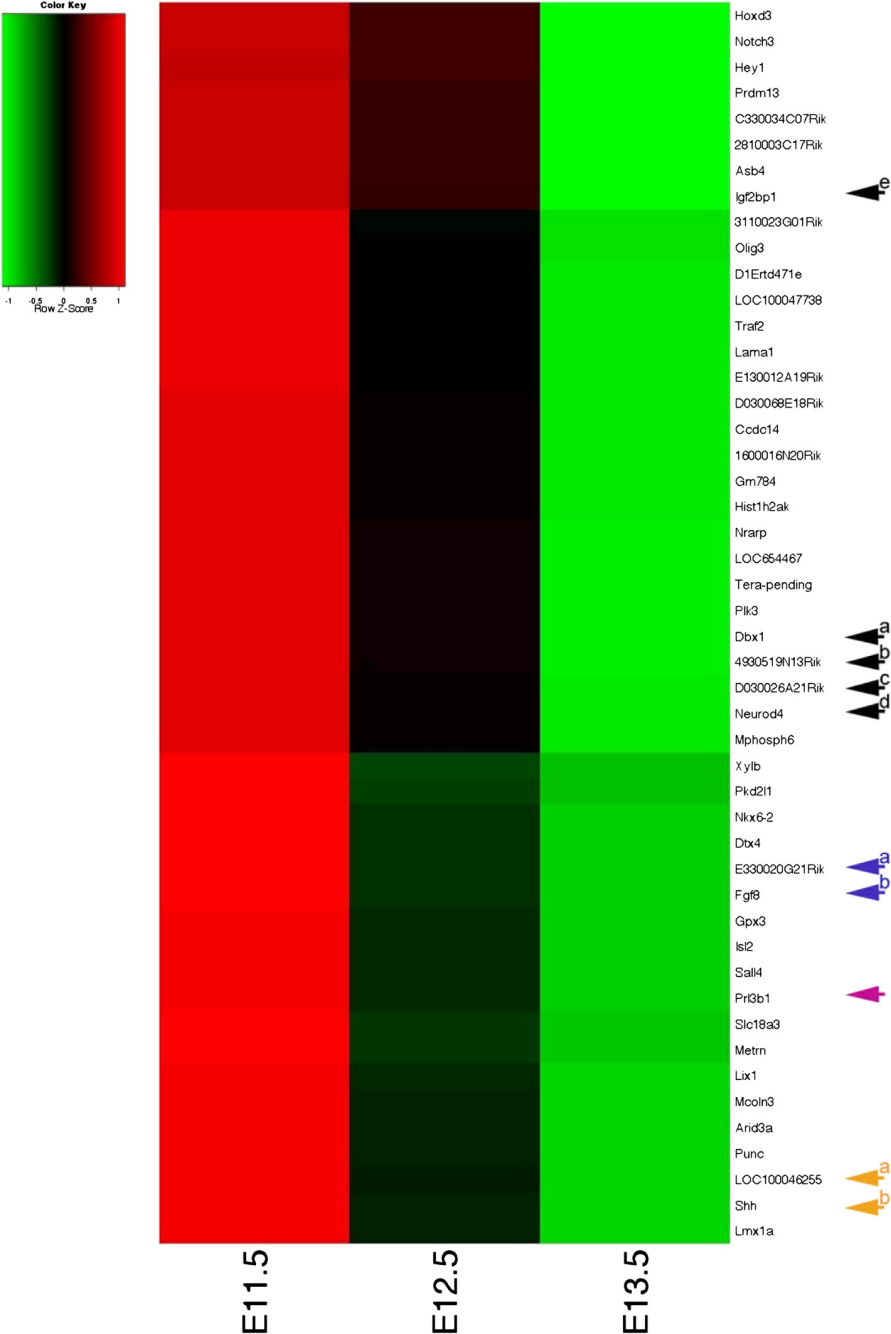
**Selected heat map profiles at E11.5, E12.5 and E13.5 of targets with known and unknown roles during mid-gestation development based on differential gene expression analysis of entire wild type embryos averaged across four strains (C57BL/6J, 129 S2/SvHsd, FVB/NHan **^**TM**^**Hsd and Hsd:ICR(CD-1)®) at any given developmental stage.** Similarities in the heat map profile can lead to the identification of new targets of interest (black arrows a to d, blue arrows, orange arrows), suggest relevant stages to investigate further (purple arrow, black arrow “b”) or possible splice variants (black arrows “c” and “d”).

**Figure 3:**
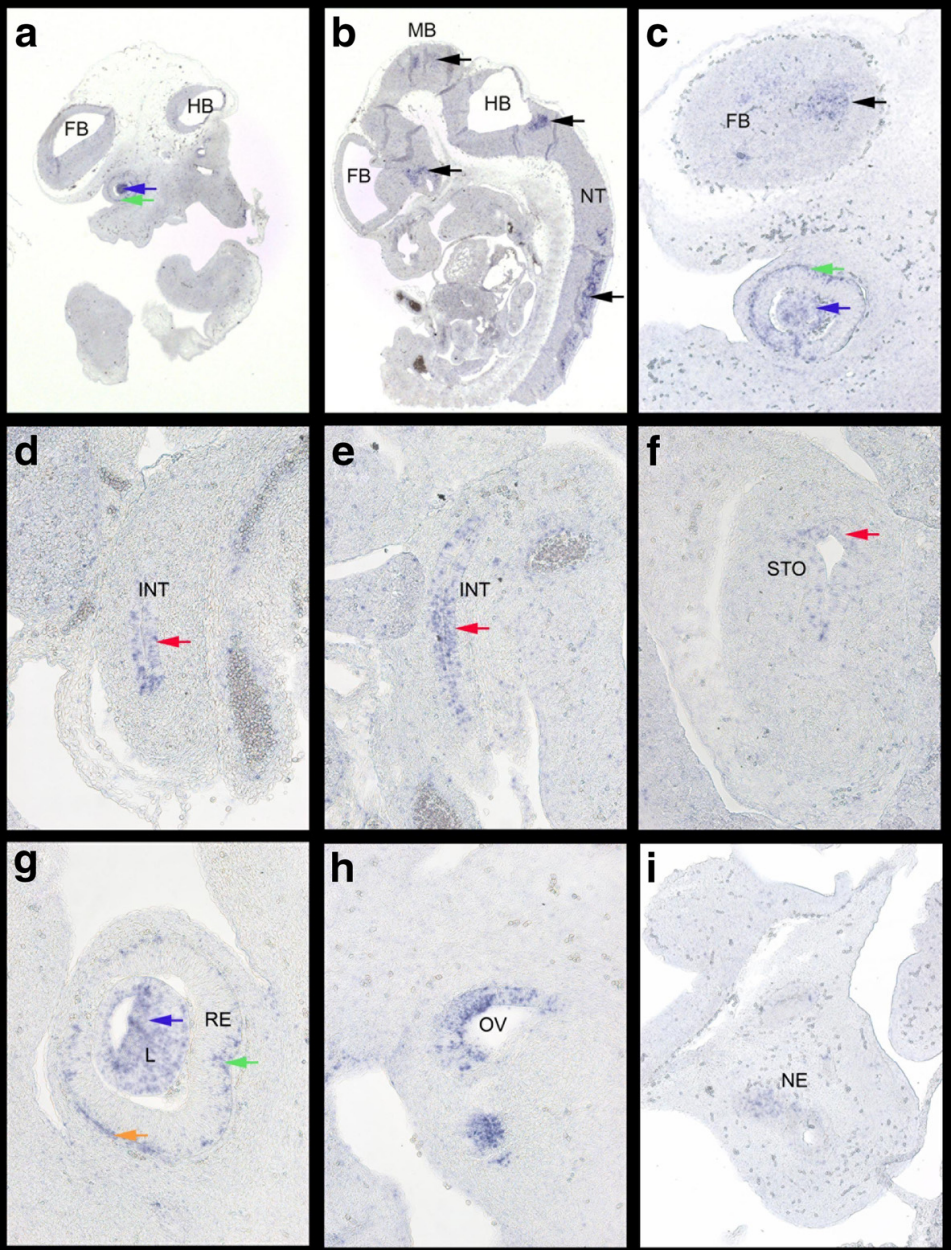
***Prl3b1 *expression at E11.5 visualized by RNA-ISH on sagittal sections of Hsd:ICR(CD1)® wild type embryos. Parasagittal (a) lateral and (b) medial section through the embryo at 32x showing expression in forebrain (FB), midbrain (MB), hindbrain (HB) and neural tube (NT) (black arrows), the lens (blue arrow) and retina (green arrow) (c) Parasagittal lateral section through forebrain and eye at 100x showing expression in the forebrain (FB, black arrow), lens (blue arrow) and retina (green arrow). (d/e/f)** sections through stomach (STO) and gut (INT) at 200x with expression in the epithelial layers (red arrow). **(g/h)** Parasagittal sections through the eye (neural layer (green arrow) and pigment layer (orange arrow) of the retina (RE), lens (L, blue arrow) and otic vesicle (OV) respectively at 200x and **(i)** through the nasal region at 100× showing expression in the nasal epithelium (NE).

**Figure 4:**
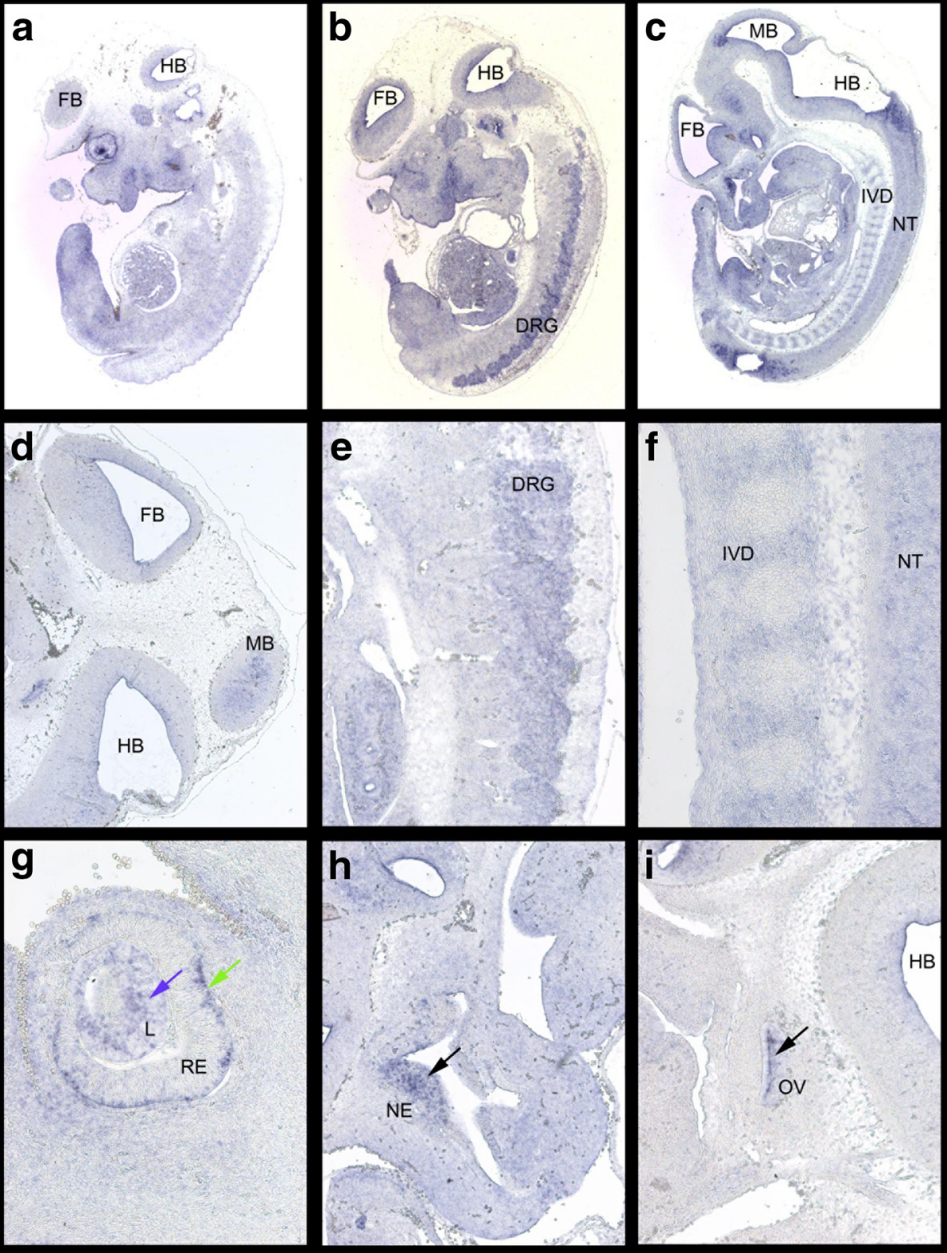
***Ninl *expression at E11.5 visualized by RNA-ISH on parasagittal sections of Hsd:ICR(CD1)® wild type embryos. (a/b/c)** Sections through the embryo from parasagittal lateral to medial at 32× showing expression in the forebrain (FB), midbrain (MB) and hindbrain (HB), the neural tube (NT), the sensory organs, the dorsal root ganglia (DRG) and the intervertebral discs (IVD). **(d)** Section through the head at 50× showing expression in forebrain (FB), midbrain (MB) and hindbrain (HB). **(e)** latero-medial section through dorsal root ganglia at 100×. **(f)** Medial section through the vertebral column at 200× showing expression in the intervertebral disc (IVD) and neural tube (NT). **(g)** Section through the eye at 200× showing expression in the lens (L, blue arrow) and retina (RE, green arrow). **(h/i)** sections through the nasal epithelia (NE) and otic vesicle (OV), respectively, at 100× (black arrows).

We chose these three stages because a switch in the developmental program from predominantly embryonic patterning at E11.5 to embryonic growth and organogenesis by E13.5 would be expected, being aware that this will reflect total expression of transcripts within the embryo and not tissue specific changes. Harboring this in mind, indeed, the anticipated gross change in gene expression during normal embryonic development was reflected in the gene expression profile obtained from our microarray data, with known patterning genes like for example *Shh* or members of the *Hox* and *Msx* families found in the group of probes that reflect decreased expression, while genes encoding structural proteins such as proteoglycans, collagens and keratins reside in the group of probes reflecting increased expression (see Additional file 4: Figure S1). This reflection of known and anticipated gene expression profiles over embryonic mouse development previously established by robust methods like RNA in-situ hybridization (RNA-ISH) not only strengthened our confidence in the data generated by the Illumina MouseWG-6_V2_0_R3_11278593 array, it also allowed us to screen for genes with similar expression profiles but a previously unknown function on a broader scale than would be possible by the rather labor intensive RNA-ISH. More as a proof of principle rather than with any specific interest in any developmental progress in particular, we randomly picked some still not annotated genes for further investigation. We chose *E330020G21Rik* (see Figure 2, Blue Arrow “a”), a so far unidentified probe in the MGI database which shared a time course heat map profile similar to *Fgf8* in our array analysis (see Figure 2, Blue Arrow “b”). A subsequent BLAST http://mouseblast.informatics.jax.org search against the murine genome showed 100% sequence identity with *Fli1* described as transiently expressed during mouse embryogenesis [40]. For another probe similar to this group in our time course heat map profile, *Prl3b1* (see Figure 2, Purple Arrow) or *Prolactin family 3, subfamily b, member 1* (MGI:97607) RNA-ISH has previously been carried out at E8.5, E10.5 and E14.5 according to the MGI database. Expression was described in the urogenital system for whole mount RNA-ISH at E10.5 [41] and during placental development for section RNA-ISH at E8.5 and E14.5 [42]. Based on the information from our microarray analysis expression E11.5 appeared a stage worth investigating. Indeed, section-ISH on E11.5 wild type Hsd:ICR(CD-1)® embryos showed distinct expression of *Prl3b1* in the CNS, the neural tube (Figure 3B) and the forebrain (Figure 3C), in the developing eye (Figures 3A,C,G), specifically the lens and retina, in the inner ear (Figure 3H) and the nasal epithelium (Figure 3I), further in the epithelial layer of intestine (Figures 3D,E) and stomach (Figure 3F). *LOC100046255* (see Figure 2, Orange Arrow “a”) a still unidentified probe according to the MGI database, yet of interest because it is sharing a heat map profile with *Shh* (see Figure 2, Orange Arrow “a”) in our time course heat map and was classified as similar to a homeobox protein according to Illumina, could subsequently be identified in a BLAST search as *Msx2*, a known and important player during mouse embryonic development [43]. Similarly *4930519N13Rik* (see Figure 2, Black Arrow “b”) also known as *Ninein-like* (*Ninl*, MGI:1925427) shared a similar heat map profile with *Dbx1* (*developing brain homeobox 1,* MGI: 94867, PMID: 7811640 + 8798145, see Figure 2, Black Arrow “a) and *D030026A21RIK* (see Figure 2, Black Arrow “c”) also known as *Insulin-like growth factor 2 mRNA binding protein 1* (*Igf2bp1*, MGI: 1890357) is sharing a heat map profile similar to *Neurod4* (*neurogenic differentiation 4*, MGI:108055, PMID 16602821) see Figure 2, Black Arrow “d”). For *Ninl* so far only regionally restricted weak to moderate expression in the liver is described by the Eurexpress database http://www.eurexpress.org at E14.5. Based on our heat map profile E11.5 appeared to be a stage worth further investigation (see Figure 2). Section-ISH on E11.5 wild type Hsd:ICR(CD-1)® embryos showed signals in the CNS (brain and neural tube) (Figures 4B,C,D,F) and the dorsal root ganglia (Figure 4E). There is also expression in the developing sensory organs, the eye (Figures 4A,G), the olfactory epithelia (Figure 4H) and the otic vesicle (Figure 4I). Of note, while the tendency of published *Igf2bp1* expression [44] is reflected in our heat map for both *Igf2bp1*, which is represented as its own probe on the array, and *D030026A21RIK* a second probe for *Igf2bp1* (see Figure 2, Black Arrow “c”) both show a similar but not identical profile in the array (see Figure 2, Black Arrow “e”) which could be attributed to the different regions of the probe design, reflecting the presence of alternative splice variants for this gene [45] or stages of RNA editing similar to what we have observed for transcripts detected by the “*Cfl1* probe” on this array.

### The effect of a decrease in tissue diversity and an increase in genetic background diversity on the outcome of microarray based gene expression profiling

While only a small number (<2%) of probes did show significant (FC >1.5) differences in their expression profile at a given time point when subjecting entire embryos to a microarray based strain by strain gene expression profiling, there was concern that the expression profiles of some genes particularly those with multiple roles during development might have been “diluted” by subjecting entire embryos to microarray gene expression analysis without prior enrichment of the target tissue. To address the impact of a decrease in tissue diversity and/or increase in genetic background diversity on the outcome of microarray based gene expression profiling and hence to address the possibility of a “diluted” expression profile when performing a differential gene expression analysis of entire wild type embryos, in a separate study, we subjected E12.5 eviscerated embryos of 11 different commonly used strains in mouse genetics, immunological studies, transgenic and gene targeting approaches [129 S2/SvHsd, FVB/NHan ^TM^Hsd, C3H/HeNHs, CBA/JHsd, BALB/cOlaHsd, C.B-17/IcrHan^TM^Hsd-*Prkdc*^*scid*^, C57BL/6J, B6;SJL-Tg(Col2a1-cre)1Bhr/J, 129 S4/SvJaeSor-*Gt(Rosa)26Sor*^*tm1(FLP1)Dym*^/J, C57BL/6-Tg(Zp3-cre)93Knw/J, Hsd:ICR(CD-1)®] to differential gene expression profiling. The E12.5 embryos were staged by the same stringent morphological criteria and then eviscerated, limiting our analysis essentially to the developing neuro/sensory, skeletal and muscular tissue. We followed the same basic experimental design as described earlier with four biological replicates per strain profile (for details refer to Experimental Design in the Material and Methods section). The pooling of three embryos per biological replicate should minimize any expression differences related to the evisceration procedure. We then conducted our analysis in two ways: First, we focused on the four strains common between this study and our previous one on entire embryos, comparing the lists of differentially regulated genes at E12.5 to address to what extent a decrease in tissue diversity impacts on differential gene expression profiling. Second, we included all 11 strains of only the eviscerated embryos at E12.5 in the analysis to see the impact of an increase in strain diversity on differential gene expression profiling. From these comparisons, we made four major observations:

Firstly, a list of 503 targets (1.11% of total probes on the array) with a fold change (FC) >1.5 derived from a strain by strain comparison of the expression profile of entire embryos at E12.5 and a list of 3403 targets (7.5% of total probes on the array) with a FC >1.5 derived from a strain by strain comparison of the expression profile of eviscerated embryos at E12.5 could be identified and ranked by total FC across the four wild type strains analyzed (129 S2/SvHsd, FVB/NHan^TM^Hsd, C57BL/6J, B6;Hsd:ICR(CD-1)®). (For details regarding the probe ranking refer to Experimental Design in the Material and Methods section.) We could identify eight of our top 50 targets established for the expression profile of entire E12.5 wild type embryos in the top ten targets of the list generated for eviscerated E12.5 wild type embryos [See Table 1 – (Rank A) Gene Name (Rank B)]: (3) *Sspn* (9), (8) *C920006O11Rik* (5), (9) *Lrrc57* (7), (10) *Fcer1g* (2), (11) *Ctse* (10), (19) *Tm7sf3* (1), (21) *Pou6f1* (4) and (25) *LOC100041516* (3). Similarly we located nine of our top 50 targets for the eviscerated embryos in the top ten ranking targets of a list generated for the expression profile of entire embryos [46][See Table 1 – (Rank B) Gene Name (Rank A)]: (2) *Fcer1g* (10), (5) *C920006O11Rik* (8), (7) *Lrrc57* (9), (9) *Sspn* (3), (14) *LOC382555* (1), (15) *Tmem87a* (6), (34) *Cap1* (7), (43) *Gramd4* (2) and (49) *EG384179* (5). For further details see Table 1 and Additional file 2: Table S2 and Additional file 5: Table S4.

Secondly, addressing the aspect of a “diluted” expression profile: Only 50% of the top 50 ranking targets in the list of E12.5 eviscerated embryos could be found to be significantly differentially expressed (FC > 1.5) between the four strains at E12.5 when subjecting the entire embryo to this analysis, while 41 of the 50 top ranking targets (82%) of the 503 targets listed for the entire E12.5 wild type embryos would be detected as significantly differentially expressed in the list of eviscerated E12.5 embryos (limited to the top 500 targets for comparability), suggesting that some differential expression was indeed lost through “dilution” if the tissue is too heterogeneous and/or the target is naturally expressed in multiple tissues of the embryo. Hence the purer the tissue type analyzed, the more likely all relevant targets can be identified with current array based gene expression profiling, making the ideal source a combination of tissue micro dissection along with sorting of gene specific fluorescence labeled cells (Lufkin Lab, work in progress).

Thirdly, addressing the aspect of an increase in strain diversity by looking at eviscerated wild type embryos at E12.5 only, now including all 11 strains: A list of 8805 targets (19% of total probes on the array) with a fold change (FC) >1.5 between any two strains could be derived from a strain by strain comparison with *Tm7sf3* (1), *Fcer1g* (2), *C920006O11Rik* (3), *Lrrc57* (4), *Pou6f1*(7), *Sspn* (8) and *LOC382555* (9) still being found in the top ten targets when ranked by total fold change (See Table 2 and Additional file 6: Table S5).

**Table 2:** Panther gene ontology (GO) analysis showing the percentage of the number (#) of genes for each GO term based on the total number of genes for each of the three data sets analyzed

**Data set**	**(A) E12.5 entire embryos/4 strains**		**(B) E12.5 eviscerated embryos/4 strains**		**(C) E12.5 eviscerated embryos/11 strains**	
GO Categories	# genes	% total	# genes	% total	# genes	% total
apoptosis (GO:0006915)	7	2.4	109	5.3	287	5.7
cell adhesion (GO:0007155)	14	4.7	213	10.3	524	10.4
cell communication (GO:0007154)	60	20.3	625	30.3	1508	30
cell cycle (GO:0007049)	30	10.1	206	10	541	10.8
cellular component organization (GO:0016043)	14	4.7	183	8.9	401	8
cellular process (GO:0009987)	92	31.1	831	40.3	2011	40
developmental process (GO:0032502)	34	11.5	450	21.8	1047	20.8
generation of precursor metabolites and energy (GO:0006091)	5	1.7	27	1.3	75	1.5
homeostatic process (GO:0042592)	4	1.4	27	1.3	56	1.1
immune system process (GO:0002376)	32	10.8	370	18	892	17.7
localization (GO:0051179)	1	0.3	7	0.3	24	0.5
metabolic process (GO:0008152)	154	52	935	45.4	2345	46.6
regulation of biological process (GO:0050789)	1	0.3	6	0.3	23	0.5
reproduction (GO:0000003)	15	5.1	91	4.4	216	4.3
response to stimulus (GO:0050896)	21	7.1	237	11.5	585	11.6
system process (GO:0050896)	27	9.1	340	16.5	774	15.4
transport (GO:0006810)	37	12.5	377	18.3	882	17.5
Total # genes	296	n/a	2060	n/a	5027	n/a
Total # processed hits	548	n/a	5034	n/a	12191	n/a

**A** (entire embryos of four strains (C57BL/6J, 129 S2/SvHsd, FVB/NHan ^TM^Hsd and Hsd:ICR(CD-1)® at E12.5), **B** (eviscerated embryos of four strains (C57BL/6J, 129 S2/SvHsd, FVB/NHan ^TM^Hsd and Hsd:ICR(CD-1)® at E12.5) and **C** (eviscerated embryos of 11 strains (129 S2/SvHsd; FVB/NHan ^TM^Hsd; C3H/HeNHs; CBA/JHsd; BALB/cOlaHsd; C.B-17/IcrHan^TM^Hsd-*Prkdc*^*scid*^; C57BL/6J; B6; SJL-Tg(Col2a1-cre)1Bhr/J; 129 S4/SvJaeSor-*Gt(Rosa)26Sor*^*tm1(FLP1)Dym*^/J; C57BL/6-Tg(Zp3-cre)93Knw/J; Hsd:ICR(CD-1)®) at E12.5.

**Figure 5:**
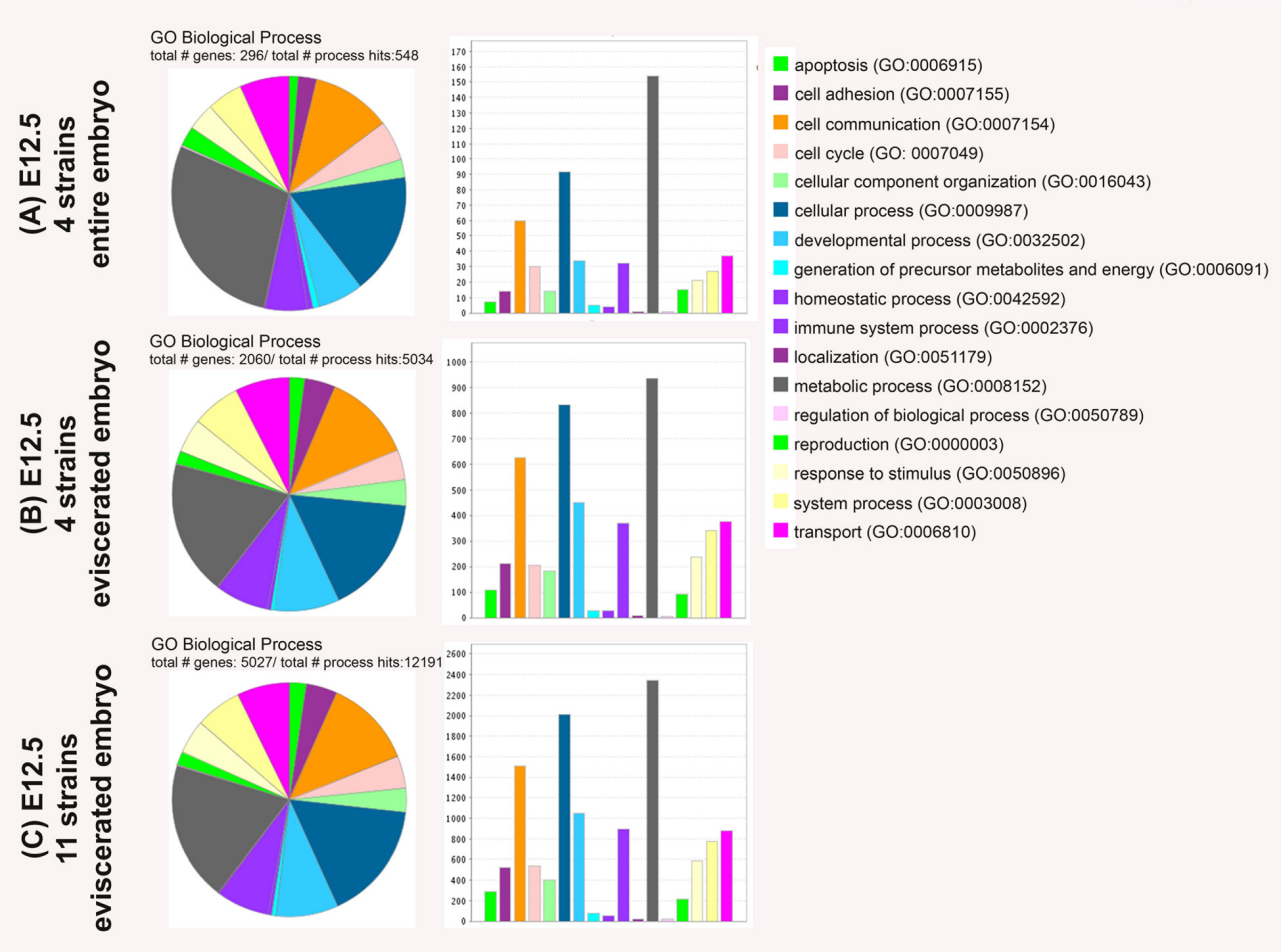
**Panther gene ontology (GO) term analysis for the three data sets (A)** (entire embryos of four strains (C57BL/6J, 129 S2/SvHsd, FVB/NHan ^TM^Hsd and Hsd:ICR(CD-1)® at E12.5), **(B)** (eviscerated embryos of four strains (C57BL/6J, 129 S2/SvHsd, FVB/NHan ^TM^Hsd and Hsd:ICR(CD-1)® at E12.5) and **(C)** (eviscerated embryos of 11 strains (129 S2/SvHsd; FVB/NHan ^TM^Hsd; C3H/HeNHs; CBA/JHsd; BALB/cOlaHsd; C.B-17/IcrHan^TM^Hsd-*Prkdc*^*scid*^; C57BL/6J; B6; SJL-Tg(Col2a1-cre)1Bhr/J; 129 S4/SvJaeSor-*Gt(Rosa)26Sor*^*tm1(FLP1)Dym*^/J; C57BL/6-Tg(Zp3-cre)93Knw/J; Hsd:ICR(CD-1)®) at E12.5 analyzed in pie view (left) and bar view (middle) alongside the color legend (right) identifying the related GO terms and numbers according to Panther.

Lastly, when subjecting the targets from each data set (set A: four strains, entire embryos/set B: four strains, eviscerated embryos/set C: 11 strains, eviscerated embryos) to a Panther Gene Ontology analysis www.pantherdb.org/panther/ontologies.jsp no loss or addition in gene ontology (GO) terms for biological based gene categories between the three data sets was observed (see Figure 5 and Table 3). When comparing the total number of genes identified in each GO term based category for biological processes for each of the three data sets, the percentage of genes classified into the GO categories for apoptosis (GO: 0006915), cell adhesion (GO: 0007155), cell communication (GO: 0007154), cellular component (GO: 0016043), cellular process (GO: 0009987), developmental processes (GO:0032502) and immune system processes (GO:0002376) increased between the data set A to the data sets B/C. While an increase in the percentage of contributing genes between data set A/B to C was limited to the GO categories localization (GO:0051179) and regulation of biological process (GO:0050789). A decrease in the percentage of contributing genes was observed for the GO term categories metabolic process (GO: 0008152) and reproduction (GO:0000003). Other categories like cell cycle (GO: 0007049), generation of precursor metabolites and energy (GO:0006091) and homeostatic process (GO:0042592) seem unaffected by the evisceration (decrease in tissue type diversity) and/or increase in genetic background diversity. 

**Table 3:** Shows the sequences of the amplification primers used for PCR

**x**	**PCR primers (5'-3')**		
**Locus**	**Forward primer**	**Reverse primer**	**Amplicon Size (bp)**
Fcer1g	5'-TTAGTGAGAGTCGAGGATCA-3'	5'-TTCCATCTCTGTAGAAAGCA-3'	480
Lipa	5'-CTCCACAGAATGTTTCTCCA-3'	5'-CCTAGTTCTCCAGAGGTTGA-3'	501
Lrrc57	5'-GTGGCTGTTGTGTTGGTGCT-3'	5'-TTGAGCCGTGTAGGTCATGG-3'	342
Gramd4	5'-CTTCTAGGCATCCTTACAGA-3'	5'-TAGACAGTTTTGTGCTCGAC-3'	446
Snx5	5'-ATGATAGTCTTCCAACTTCTC-3'	5'-TGATAGGTATTTGGATGCTG-3'	384
Zfp235	5'-GTTGACTGAAGCATCACTTC-3'	5'-TTCACCTTATTGGGTTGATA-3'	383
Sspn	5'-GTAGTAGAGTCTTCATTAAAGCC-3'	5'-TCAGTGGCTTACACACTAAG-3'	360

In summary, while a decrease in tissue diversity as well as an increase in strain diversity raises the number of probes showing a differential signal, one has to bear in mind that in a typical expression profiling study, such as the comparison of loss-of gene function versus wild type littermates or drug treated versus untreated cohorts, the actual fold changes of the identified true targets might by far outweigh the strain specific signal differences. However, the lists generated here for wild type embryos are meant to serve as guide and resource reference tool for possible gene expression profiling background noise using the Illumina Mouse WG-6 v2.0 and possibly the preceding Mouse Ref-8 v2.0 array platform in gene expression studies performed on mid-gestation embryos for strains classically used in genetic engineering.

### Differential gene expression reflects the origin of inbred strains and can serve as valuable tool to establish strain ontology relationships

Most mouse inbred strains available in laboratories today can be traced back to strains established by William Castle, Abbie Lathrop, Clarence Cook Little and Halsey J. Bagg [1,47] however, making use of polymorphisms and mutations abundant in the genome and the evolving technology, todays available inbred strains can be clearly distinguished not only by coat color but also by their DNA sequence [48-51]. Studies have shown the usefulness of a combination of quantitative trait locus (QTL), single nucleotide polymorphism (SNP) and gene expression data [46]. A paper by Petkov [52] making use of SNPs and QTL analysis displays a mouse family tree with seven distinct groups. While BALB/c, CBA and C3H substrains are all found within group 1, FVB/N in group 2, C57BL/6 in group 4 and 129 substrains in group 5 of his classification.

**Figure 6:**
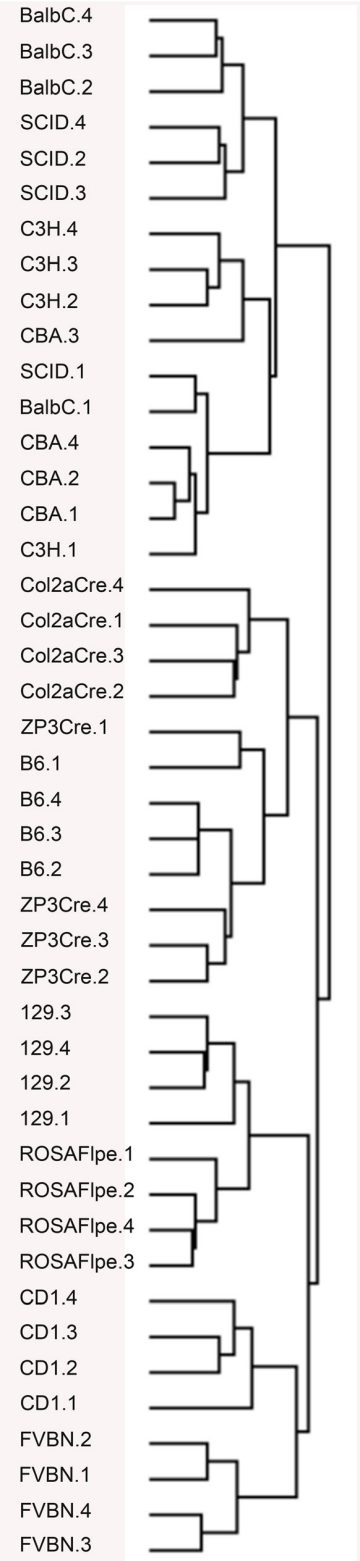
**Mouse strain gene ontology relationships for 11 strains based on microarray based differential gene expression analysis of eviscerated embryos at E12.5.** For simplicity the strain names have been abbreviated in this tree view figure. The full strain name is given in brackets preceded by the abbreviation as following: 129 [129 S2/SvHsd]; FVBN [FVB/NHan ^TM^Hsd]; C3H [C3H/HeNHs]; CBA [CBA/JHsd]; BalbC [BALB/cOlaHsd]; SCID [C.B-17/IcrHan^TM^Hsd-*Prkdc*^*scid*^]; B6 [C57BL/6J]; Col2aCre [B6; SJL-Tg(Col2a1-cre)1Bhr/J]; ROSAFlpe [129 S4/SvJaeSor-*Gt(Rosa)26Sor*^*tm1(FLP1)Dym*^/J]; ZP3Cre [C57BL/6-Tg(Zp3-cre)93Knw/J] and CD1 [Hsd:ICR(CD-1)®)]. The numbers 1 to 4 for each group refer to the biological replicate.

Here, we have subjected 11 mouse strains to microarray based differential expression analysis [129 S2/SvHsd, FVB/NHan^TM^Hsd, C3H/HeNHs, CBA/JHsd, BALB/cOlaHsd, C.B-17/IcrHan^TM^Hsd-*Prkdc*^*scid*^, C57BL/6J, B6;SJL-Tg(Col2a1-cre)1Bhr/J, 129 S4/SvJaeSor-*Gt(Rosa)26Sor*^*tm1(FLP1)Dym*^/J, C57BL/6-Tg(Zp3-cre)93Knw/J, Hsd:ICR(CD-1)®]. For the 8805 differentially expressed probes in this 11 strains comparison (19% of total probes on the array) we can find the biological replicates clustering tightly by strain origin when subjecting the data to TreeView analysis http://rana.lbl.gov/EisenSoftware.htm indicating on one side the accuracy of our assay as well as reiterating the fact that besides polymorphism on the DNA level, clear differences in gene expression have evolved for a small subset of genes in these strains and substrains (Figure 6). The clustering observed in our study is supported by the SNP and QTL based study [52]. Based on array clustering, we can define two major groups of mouse strains in our study:

I BALB/cOlaHsd, C.B-17/IcrHan^TM^Hsd-*Prkdc*^*scid*^, CBA/JHsd and C3H/HeNHs.

II B6;SJL-Tg(Col2a1-cre)1Bhr/J, C57BL/6-Tg(Zp3-cre)93Knw/J, C57BL/6J, 129 S2/SvHsd, 129 S4/SvJaeSor-*Gt(Rosa)26Sor*^*tm1(FLP1)Dym*^/J, Hsd:ICR(CD-1)® and FVB/NHan^TM^Hsd.

Within group II we can further subdivide between Group IIa, containing B6;SJL-Tg(Col2a1-cre)1Bhr/J, C57BL/6-Tg(Zp3-cre)93Knw/J, C57BL/6J all comprising a C57BL/6 genetic background, with B6;SJL-Tg(Col2a1-cre)1Bhr/J initially being generated on the SJL background and subsequently mated onto a C57BL/6J background and Group IIb represented by Group IIb-1 129 S2/SvHsd, 129 S4/SvJaeSor-*Gt(Rosa)26Sor*^*tm1(FLP1)Dym*^/J both of 129 genetic background and Group IIb-2 represented by Hsd:ICR(CD-1)® on one side and FVB/NHan^TM^Hsd on the other side of the array clustering branch. ICR mice, an outbred strain, had not been subjected to Perkov’s study [52].

Unlike previous studies, where strain relationships have been established on the DNA level or combinations of DNA and gene expression analysis [46,52], we demonstrate here that the function driven analysis of microarray gene expression profiling is sufficient for the accurate confirmation of the genetic ancestry of mouse strains.

## Conclusions

Since the results of microarray gene expression profiling can be impacted on by variations in the strain of mouse used, we aimed to provide a resource reference list of probes contributing to strain differences or “noise” when subjecting non-isogenic tissue from any of the frequently used inbred strains in mouse gene targeting or even an outbred strain, to microarray based differential gene expression profiling using the Illumina Mouse WG6 v2.0 microarray chip. We subjected entire embryos as well as eviscerated embryos to this study. This reduction in tissue diversity was reflected in a raised number of significantly differentially expressed genes, a number likely to increase with further reduction of tissue heterogenity. The data retrieved from our extensive expression profiling using the Illumina platform is robust and reflects the anticipated gene expression patterns of known and well-characterized patterning and structural genes during mid-gestation development. For a small number of probes, the data has been impacted on by probe design artifacts (probes not allocated in the actual gene) or natural genetic polymorphisms between mouse strains reflecting “background noise”. This is a problem researchers should be aware of and future array platforms would need to adjust for in order to be a reasonable experimental choice compared to the rapidly evolving RNA-seq technology, which, once affordable, should allow for a more unbiased expression profiling analysis in the near future (see review by [10]).

At the current point in time, owing to the overall robustness of the array, heat map profile similarities make way for the discovery of genes with previously unknown function. Lastly, microarray based differential gene expression analysis on its own can serve as a tool to establish strain ontology relationships similar to SNP and QTL analysis.

## Methods

### Experimental design

All embryos were carefully staged according to morphological criteria [53] and only embryos showing all morphological criteria as displayed for each stage E11.5, E12.5 and E13.5 were included in this study. Three age-matched embryos of undefined gender were combined to form one biological replicate. Altogether a total of four biological replicates (12 embryos) were analyzed for each strain and developmental time point and subjected to expression profiling using the MouseWG-6_V2_0_R3_11278593 array from Illumina. We chose this approach of embryo pooling according to the experimental design by Korostynski et al. [20] to minimize the contribution of individual differences or slight technical variations from embryo dissections/eviscerations to the read out of the differential expression analysis. The fold change (FC) of expression for the four samples per strain was averaged at each of the three time points and subjected to a strain-by-strain comparison for a given developmental stage. The genes were then ranked according to the highest total fold change across all strains. Only genes with a FC >1.5 between any of the strains were considered as significantly differentially expressed.

### Ethics statement, mouse husbandry and tissue collection

All animal procedures were performed according to the Singapore A*STAR Biopolis Biological Resource Center (BRC) Institutional Animal Care and Use Committee (IACUC) guidelines and the IACUC protocols employed were reviewed and approved by the aforementioned committee before any animal procedures were undertaken for this study described here (IACUC Protocol No: 080348 and 080377). The mouse strains used in this study were maintained and provided by the A*STAR Biopolis Biological Resource Center (129 S2/SvHsd, FVB/NHan^TM^Hsd, C3H/HeNHsd, CBA/JHsd, BALB/cOlaHsd and C.B-17/IcrHan^TM^Hsd-*Prkdc*^*scid*^, C57BL/6J, Hsd:ICR(CD-1)®) or directly imported from Jackson Laboratories (B6;SJL-Tg(Col2a1-cre)1Bhr/J #003554, 129 S4/SvJaeSor-*Gt(Rosa)26Sor*^*tm1(FLP1)Dym*^/J #003946, C57BL/6-Tg(Zp3-cre)93Knw/J #003651) and then maintained according to Jackson Laboratories guidelines specific for each strain. Males and females of each respective strain were intermated to generate E11.5, E12.5 and E13.5 embryos, with E0.5 being defined as the day the vaginal plug was detected. The mouse embryos were subsequently harvested in ice-cold Leibovitz’s L-15 medium (Gibco) at E11.5, E12.5 or E13.5 and critically staged applying morphological criteria as described [53]. For the study focusing on differential gene expression in eviscerated embryos the E12.5 embryos were dissected free of all internal organs. Embryos were then dissociated for RNA isolation.

### RNA extraction and microarray analysis

Fresh mouse embryonic tissues were rapidly dissociated into small clumps in L-Leibovitz medium by repeated pipetting. The small tissue clumps were collected by centrifugation at 2000 rpm for 5 minutes. Applying the TRIzol/RNeasy hybrid method, TRIzol (Invitrogen) was added to the pelleted tissues at approximately 1 ml per 50 mg tissue for homogenization. The homogenate was stored in −80°C for no longer than 3 months before RNA extraction. During the RNA extraction, 0.2 ml chloroform was added per 1 ml of homogenate and the top aqueous phase was gained after centrifugation at 12,000 g for 15 minutes at 4°C. The aqueous phase was loaded onto a gDNA Eliminator spin column based on the DNA removal capacity of the column. Subsequent steps were done according to the RNeasy Plus Mini kit (Qiagen) following the manufacturer’s instruction. Total RNA extracted from fresh mouse embryonic tissues was quantified by a NanoDrop ND-1000 Spectrophotometer. For quality control, RNA was diluted to the working concentration of the Agilent RNA 6000 Nano Kit and 1 ul of the diluted RNA sample was run on the Nano chip using an Agilent 2100 electrophoresis Bioanlyzer. The Nano chip assay was performed according to the manufacturer’s instructions. The quality of total RNA was assessed primarily via the profile of the electropherogram and secondarily by RNA integrity number (RIN) generated by the Bioanalyzer software, only samples with a RIN > 9.4 were included in the study (see Additional file 5: Table S4). The RNA concentration given by Nanodrop and Nano chip coincided. For each biological replicate 50 ng of high quality total RNA was labeled using Illumina TotalPrep RNA Amplification kit from Ambion and hybridized on Illumina’s MouseWG-6_V2_0_R3_11278593 array according to the manufacturer’s instructions. Microarray data was normalized using GenomeStudio (background subtraction, rank invariant normalization). Any negative values were replaced by the value “1” for fold change calculation and then all signals were Log2 transformed. Linear modeling of the transformed data was performed using Limma in R [54] with the Benjamini and Hochberg correction. The model used included developmental stage, strain and batch factors, where appropriate. P value and FDR were obtained for coefficient of each factor depending on the comparison, ie. coefficients for developmental stage or strain. Fold difference was calculated by taking the ratio of the individual signals with higher expressing value over the signals with the lower expression value. Only expression levels with at least 1.5× fold difference and a false discovery rate (FDR) below 5% were considered as significantly differentially regulated. Microarray data was hierarchical clustered by average linkage clustering with uncentered correlation using Cluster [55] and the heatmap was generated with R. Strain ontology relationships were established with TreeView http://rana.lbl.gov/EisenSoftware.htm.

### Genomic DNA extraction and sequencing over illumina probes

To extract genomic DNA embryos were removed from the yolk sac, briefly washed in 1x PBS and placed in 500 ul PKDB digestion buffer (50 mM Tris–HCl (pH 8.0), 200 mM NaCl, 5 mM EDTA, 1% SDS) containing 1 mg/ml proteinase K and incubated at 55°C overnight. Digested samples were extracted with an equal volume of phenol-chloroform, DNA was precipitated with ethanol and washed with 70% ethanol. DNA pellets were air-dried and resuspended in DNase free water.

Sequences 500 nucleotide upstream and downstream of each Illumina probe sequence were obtained from UCSC using BLAST-Like Alignment Tool (BLAT) at ( http://genome.ucsc.edu/). For primer design, sequences obtained from BLAT were imported into the Vector NTI Advance 10 (Invitrogen, CA, USA) software and primer pairs flanking the Illumina probe sequences were designed carefully avoiding similarities with repetitive sequences or other loci in the genome. Table 3 shows the sequences of the amplification primers used for PCR.

PCR products were generated using Platinum® Pfx DNA polymerase (Invitrogen) and PCR products were purified on a MinElute PCR purification spin column (Qiagen, Hagen, Germany) following the manufacturer's instructions. The DNA was eluted in 30 ul of elution buffer and sent for sequencing. Sequences were aligned against and compared with the sequences of respective Illumina probes using Vector NTI .

### cDNA synthesis and real-time qPCR analysis

Total RNA from mouse embryos was isolated, assessed and quantified as described above. First strand cDNA was synthesized from 5ug of total RNA by reverse transcription PCR at 50°C for 30 min in the presence of 200 ng/ul random hexamers and 10 mM each of dNTPs and RevertAid™ Premium Enzyme mix (Fermentas). The synthesized cDNAs were adjusted to 50 ng/ul of which 100 ng was used in a final volume of 20 ul. Each sample was run in triplicate on an Applied Biosystems 7500 Real-Time PCR systems using Maxima™ SYBR Green/ROX qPCR master mix (Fermentas). The *Hprt* gene had no significant variation in expression across the four mouse strains and therefore was used as endogenous control for normalization . Expression level was evaluated relative to a calibrator according to the 2^-ΔΔCt^ method for quantitation.

### Histology and RNA in situ hybridization

Mouse embryos were processed by fixation with 4% paraformaldehyde (PFA), overnight at 4°C, then washed with 1x PBS, dehydrated in graded ethanol and embedded in paraffin. A Leica RM 2165 microtome was used to make 10 um thick paraffin sections. Sectioned in situ hybridization was performed as described in [56]. The cDNA of 0.8 kb *Prl3b1* (IMAGE clone: 30787415) linearized with *EcoRV* and *Ninl* cDNA of 4.2 kb (IMAGE clone: 30615484) linearized with *EcoRI* were used as templates for synthesizing antisense DIG-labeled *Prl3b1* and *Ninl* RNA probes (DIG RNA labeling kit, Roche).

## Competing interests

The authors declare that they have no competing interests.

## Authors' contributions

All authors contributed to drafting the manuscript. PK, YSP and TL were involved in the design of the study. PK interpreted the data and carried out the animal work. XX generated the microarray data. XX and SLL carried out the validation of microarray results. VS carried out RNA-ISH. MEF, HL and RKMK carried out computational analysis of the microarray data. All authors have read and approved the final manuscript.

## Supplementary Material

Additional file 1 **Table S1.** List of significant differentially regulated probes with a fold change >1.5× for entire embryos of four strains (C57BL/6J, 129 S2/SvHsd, FVB/NHan ^TM^Hsd and Hsd:ICR(CD-1)®) at E11.5 subjected to microarray expression analysis. Expression values are included. Click here for file

Additional file 2 **Table S2.** List of significant differentially regulated probes with a fold change >1.5× for entire embryos of four strains (C57BL/6J, 129 S2/SvHsd, FVB/NHan ^TM^Hsd and Hsd:ICR(CD-1)®) at E12.5 subjected to microarray expression analysis. Expression values are included. Click here for file

Additional file 3 **Table S3.** List of significant differentially regulated probes with a fold change >1.5× for entire embryos of four strains (C57BL/6J, 129 S2/SvHsd, FVB/NHan ^TM^Hsd and Hsd:ICR(CD-1)®) at E13.5 subjected to microarray expression analysis. Expression values are included. Click here for file

Additional file 4 **Figure S1.** Heatmap displaying all significant differentially regulated probes with a fold change >1.5× for entire embryos of four strains (C57BL/6J, 129 S2/SvHsd, FVB/NHan ^TM^Hsd and Hsd:ICR(CD-1)®) at E11.5, E12.5 and E13.5 subjected to microarray expression analysis. Click here for file

Additional file 5 **Table S4.** List of significant differentially regulated probes with a fold change >1.5× for eviscerated embryos of four strains (C57BL/6J, 129 S2/SvHsd, FVB/NHan ^TM^Hsd and Hsd:ICR(CD-1)®) at E12.5 subjected to microarray expression analysis. Click here for file

Additional file 6 **Table S5.** List of significant differentially regulated probes with a fold change >1.5× for eviscerated embryos of 11 strains (129 S2/SvHsd; FVB/NHan ^TM^Hsd; C3H/HeNHs; CBA/JHsd; BALB/cOlaHsd; C.B-17/IcrHan^TM^Hsd-*Prkdc*^*scid*^; C57BL/6J; B6; SJL-Tg(Col2a1-cre)1Bhr/J; 129 S4/SvJaeSor-*Gt(Rosa)26Sor*^*tm1(FLP1)Dym*^/J; C57BL/6-Tg(Zp3-cre)93Knw/J; Hsd:ICR(CD-1)®). at E12.5 subjected to microarray expression analysis. Click here for file
